# Methicillin-resistant Staphylococcus aureus mandibular osteomyelitis in an extremely low birth weight preterm infant

**DOI:** 10.1186/s13052-015-0163-1

**Published:** 2015-08-04

**Authors:** Silvia Martini, Fabio Tumietto, Rita Sciutti, Laura Greco, Giacomo Faldella, Luigi Corvaglia

**Affiliations:** Neonatal Intensive Care Unit, Department of Medical and Surgical Sciences, St. Orsola-Malpighi Hospital, University of Bologna, Bologna, Italy; Infectious Diseases Unit, Department of Medical and Surgical Sciences, St. Orsola-Malpighi Hospital, University of Bologna, Bologna, Italy; Pediatric Radiology Operative Unit, Department of Medical and Surgical Sciences, St. Orsola-Malpighi Hospital, University of Bologna, Bologna, Italy

## Abstract

Methicillin-resistant Staphylococcus aureus (MRSA) is an established nosocomial pathogen with frequent multidrug resistance. The immaturity of the immune system along with intravascular lines and empirical antibiotic treatments place hospitalized preterm infants at major risk of MRSA infection.

We report a case of MRSA mandibular osteomyelitis complicating a persistent S. aureus bacteremia in a 23-week preterm infant. From the first weeks of life, the infant showed recurrent C-reactive protein (CRP) elevation, associated with S. aureus bacteremia. Antibiotic courses, including vancomycin and linezolid, were performed with transitory normalization of blood parameters. On day 74, the infant suddenly deteriorated and showed a significant increase of both CRP and procalcitonin. Empiric vancomycin and piperacillin-tazobactam treatment was started; nevertheless, she developed a progressive hard swelling of neck and mandible. Radiological evaluation revealed a mandibular osteomyelitis complicated by an abscess, whose culture grew MRSA. Vancomycin was thus changed to teicoplanin and complete clinical and radiological healing was gradually achieved.

In the presence of major risk factors, persistent bacteremia and nonspecific symptoms, a localized focus of infection should be suspected. Microbiological diagnosis should always be attempted and antibiotic treatment should be guided by both susceptibility results and clinical response.

## Background

*Staphylococcus aureus* is a facultative anaerobic Gram-positive bacterium. From the 1970s on, S. aureus strains resistant to beta-lactam antibiotics, called methicillin-resistant S. aureus (MRSA), have progressively emerged. These strains are typical nosocomial pathogens and often show multidrug resistance patterns.

The immaturity of the immune system, along with the widespread use of empirical antibiotic treatments, intravascular lines and other invasive devices in Neonatal Intensive Care Units, places preterm infants at great risk of MRSA infection. Colonized hospitalized neonates are 24.2 times more likely to develop an ensuing MRSA infection when compared with non-colonized [1].

We present a case of MRSA mandibular osteomyelitis complicating a persistent S. aureus bacteremia in an extremely low birth weight (ELBW) preterm infant.

## Case presentation

This 498-grams Caucasian female infant was born in April 2012 at 23 + 4 weeks of gestation by emergency cesarean section due to abruptio placentae. Apgar scores: 5–8. During the first weeks of life, she remained in critical condition; prolonged mechanical ventilation and total parenteral nutrition were required.

On day 28, the infant showed clinical deterioration and respiratory instability. Blood tests revealed a C-reactive Protein (CRP) increase (4.26 mg/dl). The ongoing percutaneous central venous catheter (PCVC) was replaced (indwelling time [IT]: 14 days) and blood cultures were performed simultaneously. Both PCVC tip and blood cultures resulted negative, whereas a real-time multiplex polymerase chain reaction (PCR) diagnostic test (SeptiFast^™^) was positive for S. aureus. According to the isolate, treatment with vancomycin (15 mg/kg twice daily) was started and continued up to day of life (DOL) 40, with subsequent CRP normalization.

At DOL 46 blood tests routinely performed showed a CRP increase (8.51 mg/dl). PCVC was replaced (IT: 18 days) and treatment with meropenem (20 mg/kg thrice daily) and vancomycin (15 mg/kg twice daily) was started. Septifast specimen was positive for S. aureus, whereas blood and PCVC tip cultures showed negative. After an initial decrease, on day 51 CRP rose again (4.41 mg/dl) and the infant developed respiratory instability with increased oxygen need. On this basis, vancomycin was changed to linezolid (10 mg/kg thrice daily). Seven days later linezolid was discontinued, whereas meropenem was maintained until DOL 69. During this period, CRP decreased and the infant improved.

On day 74 (postconceptional age 34 + 2 weeks, body weight 840 g) the infant suddenly developed lethargy, abdominal distension with bilious gastric residuals, succeeding apneas and bradycardias. Due to respiratory instability, she was put on mechanical ventilation; moreover, enteral nutrition was withheld and PCVC was replaced (IT: 8 days). Blood tests revealed an increase of both CRP (8.35 mg/dl) and procalcitonin (20.6 ng/ml). After obtaining blood cultures, empiric therapy with vancomycin (15 mg/kg twice daily) and piperacillin-tazobactam (100 mg/kg thrice daily) was started.

**Fig. 1:**
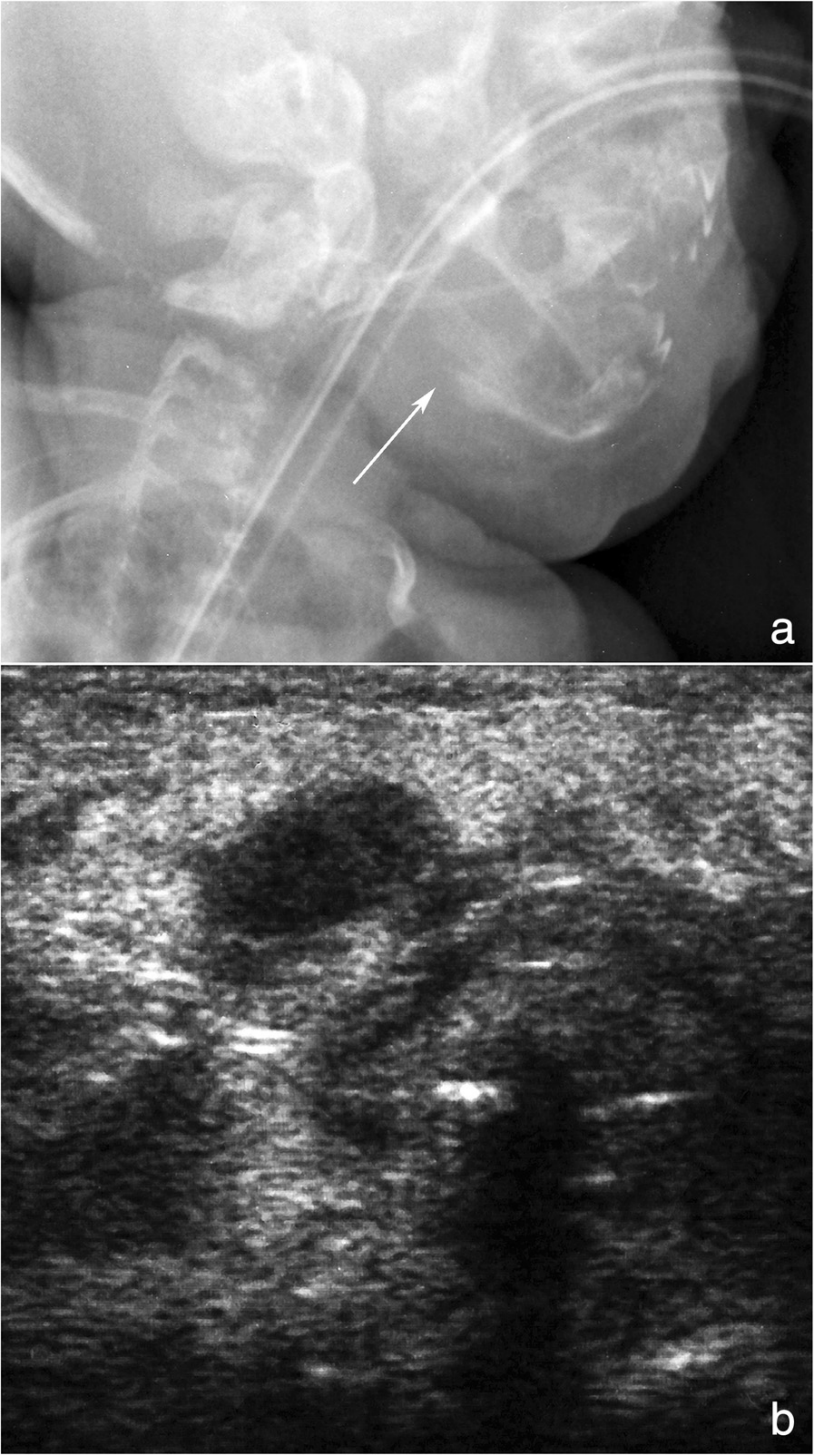
X-ray and ultrasound evaluations. Facial bone X-rays in lateral projection (**a**) showed the osteolytic disruption of the right horizontal mandibular ramus with focal periosteal reaction and swelling of the surrounding soft tissues. The ultrasound examination (**b**) confirmed X-ray findings and also demonstrated a hypo-anechoic central image consistent with initial abscess collection

Twelve hours later, the infant developed a hard swelling of neck and mandible with heat and redness of the overlying skin. Cranial X-rays revealed an osteolytic lesion of the right horizontal mandibular ramus surrounded by periosteal reaction and soft tissue swelling, consistent with osteomyelitis (Fig. 1a). Ultrasound scans confirmed the soft tissue swelling and detected the presence of a round, suppurated area (diameter 15 mm) next to the right horizontal mandibular ramus (Fig. 1b). Transthoracic echocardiography revealed no evidence of endocarditis. Blood tests were repeated, detecting CRP 23.42 mg/dl and procalcitonin 29.3 ng/ml. On this basis, antibiotic therapy with vancomycin was changed to teicoplanin (15 mg/kg IV bolus followed by 12 mg/kg after 12 and 24 hours; followed by 10 mg/kg daily) while piperacillin-tazobactam was modified to continuous infusion (400 mg/kg/24 h). A transitory leucopenia (white blood cell count 3,900/mm^3^, of which neutrophils 2,540/mm^3^ and lymphocytes 680/mm^3^) associated with piperacillin-tazobactam treatment was observed on day 76. PCVC tip and blood cultures previously sent both showed negative.

**Fig. 2:**
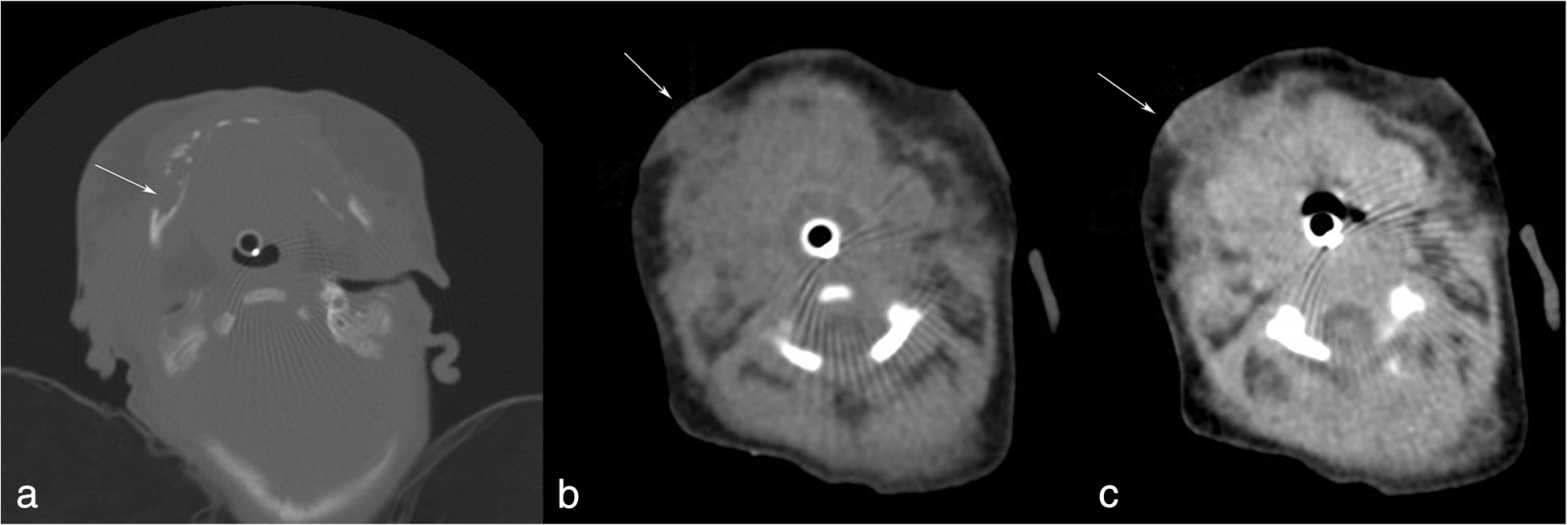
Axial computerized tomography scans. The bone window setting (**a**) confirms the large cortical bone erosion of the right mandible; the soft-tissue window setting (**b**) shows, next to the mandibular bone, a hypodense area that presents rim enhancement (**c**) after intravenous contrast medium injection, consistent with an abscess

At DOL 78 a craniofacial contrast computerized tomography (CT) scan was performed (Fig. 2). Bone window setting documented loss of structural bone integrity with cortical disruption mainly involving the right mandibular horizontal ramus. Soft-tissue window setting confirmed the inflammatory edema and showed a hypodense fluid area with peripheral contrast enhancement next to the right mandibular ramus, consistent with an abscess.

In the following hours the mandibular swelling further increased, forming a fluctuant abscess. Surgical drainage was planned; however, on day 79 the abscess spontaneously fistulated through the chin skin, draining 10 ml of purulent material, whose culture grew MRSA (minimum inhibitory concentrations [MICs]: oxacillin >2 mg/dl, penicillin >8 mg/dl, vancomycin >1 mg/L). Over the following days the infant improved and the mandibular swelling slowly reduced. CRP values gradually normalized and blood cultures remained stable negative. Continuous infusion of piperacillin-tazobactam was stopped at DOL 84 while teicoplanin was continued at the aforementioned regimen for a total of 4 weeks, according to Therapeutic Drug Monitoring plasma levels.

A near-term brain magnetic resonance imaging (MRI) scan, performed after two months, documented the complete radiological healing of both soft and bone tissues and displayed intact mandibular alveolar recesses.

The infant was discharged in good condition at DOL 165. At serial follow-up assessments (6, 12, 18 and 24 months of corrected age) no recurrence of osteomyelitis was observed and serum markers of renal function were within normal ranges. No evidence of missing/malformed mandibular teeth or delayed dental eruption was noticed.

## Discussion

Non-congenital osteomyelitis is rare in the preterm population [2–6]. Hematogenous dissemination is the leading pathophysiological mechanism; intravascular devices are identified as the main source of infection. S. aureus is the most common pathogen, with increased rates of MRSA isolates.

Periosteal reaction, lytic areas surrounded by cortical thickening and swelling of adjacent soft tissues are common radiographic findings; in the early phases, however, plain films may not reveal significant abnormalities. CT is more sensitive for early osteomyelitis diagnosis [7], being particularly effective in detecting bone sequestra. Ultrasound evaluation is a useful diagnostic tool for identifying soft tissues swelling, periosteal elevation and fluid collections next to the affected bone; unfortunately, however, a normal ultrasound scan can not rule out a diagnosis of osteomyelitis [8]. Gadolinium-enhanced MRI currently represents the gold-standard imaging technique for the early detection of osteomyelitis, complicating abscesses and related infection of extra-osseous soft tissues [9].

Clinical features of non-congenital osteomyelitis in preterm infants have been previously described [3, 6]. Systemic upset with signs of sepsis is typically present, whereas local symptoms develop later. Long bones of upper and lower extremities are mainly interested, with a high risk of long-term orthopedic sequelae. To date, however, no mandibular involvement has been reported.

In the present case, the infant required a central access over a prolonged period. Although the indwelling time of each PCVC was <20 days, it is feasible that PCVCs represented the portal entry for MRSA. The extreme prematurity and the infant’s poor weight gain, mainly due to her critical initial condition, could have further increased susceptibility to infections.

Even though the collected blood cultures were repeatedly negative, the bacterial culture of the purulent drained material allowed us to ascertain the pathogen responsible for the mandibular infection. A number of possible factors could have contributed to negative blood culture results. Firstly, from the first weeks of life this infant received repeated and prolonged antibiotic treatments. Moreover, a minimum blood volume of 1 mL is normally required for culture analysis in the neonatal population; in ELBW infants, however, low blood volume along with difficult venipuncture are a major challenge to collecting adequate blood culture specimens. At small blood volumes, PCR-based diagnostic systems seem to have a higher sensitivity when compared to blood cultures [10].

Considering the infant’s major risk factors for MRSA infection (i.e. prematurity, PCVC, previous antibiotic treatments, prolonged hospitalization), S. aureus bacteremia was firstly treated with vancomycin, which is currently the mainstay treatment for MRSA infections. Nevertheless, reports of treatment failure are increasing, particularly for vancomycin MIC > 1 mg/L [11]. Moreover, higher rates of treatment failure have also been observed in patients with MRSA bacteremia initially treated with vancomycin [12].

In the present case, vancomycin MIC of the MRSA isolated from the abscess culture was >1 mg/L (established susceptibility breakpoint: 2 mg/L). However, previous treatments with vancomycin proved to be ineffective in clearing MRSA bacteremia and preventing possible complications. We could speculate that, although the isolate showed an *in vitro* sensitivity to vancomycin, the infant did not achieve an adequate clinical response to vancomycin treatment and developed mandibular osteomyelitis. In consideration of this fact, and given the lower bone concentration of vancomycin, antibiotic treatment was finally changed to teicoplanin with subsequent clinical resolution.

## Conclusions

The extreme prematurity of the infant, along with the characteristics of isolated pathogen and the peculiarity of the infection site, contribute to the absolute rarity of the case reported. In the presence of major clinical risk factors, recurrent CRP elevation, persistent bacteremia and nonspecific symptoms, a localized focus of infection should be suspected. Microbiological diagnosis should always be attempted and antibiotic treatment should be guided by both susceptibility results and clinical response.

## Consent

Written informed consent was obtained from the patient’s parents for publication of this Case report and any accompanying images. A copy of the written consent is available for review by the Editor-in-Chief of this journal.

## Abbreviations

MRSA: Methicillin-resistant Staphylococcus aureus
ELBW: extremely low birth weight
CRP: C-reactive protein
PCVC: percutaneous central venous catheter
IW: indwelling time
PCR: polymerase chain reaction
DOL: day of life
CT: computerized tomography
MIC: minimum inhibitory concentration
MRI: magnetic resonance imaging

## References

[1] Zervou FN, Zacharioudakis IM, Ziakas PD, Mylonakis E (2014). MRSA colonization and risk of infection in the neonatal and pediatric ICU: a meta-analysis. Pediatrics.

[2] Weeks JL, Garcia-Prats JA, Baker CJ (1981). Methicillin-resistant Staphylococcus aureus osteomyelitis in a neonate. JAMA.

[3] Ish-Horowicz MR, McIntyre P, Nade S (1992). Bone and joint infections caused by multiply resistant Staphylococcus aureus in a neonatal intensive care unit. Pediatr Infect Dis J.

[4] Korakaki E, Aligizakis A, Manoura A, Hatzidaki E, Saitakis E, Anatoliotaki M (2007). Methicillin-resistant Staphylococcus aureus osteomyelitis and septic arthritis in neonates: diagnosis and management. Jpn J Infect Dis.

[5] Eggink BH, Rowen JL (2003). Primary osteomyelitis and suppurative arthritis caused by coagulase-negative staphylococci in a preterm neonate. Pediatr Infect Dis J.

[6] Williamson JB, Galasko CS, Robinson MJ (1990). Outcome after acute osteomyelitis in preterm infants. Arch Dis Child.

[7] Pineda C, Vargas A, Rodríguez AV (2006). Imaging of osteomyelitis: current concepts. Infect Dis Clin North Am.

[8] Keller MS (2005). Musculoskeletal sonography in the neonate and infant. Pediatr Radiol.

[9] Pendleton A, Kocher MS (2015). Methicillin-resistant Staphylococcus aureus Bone and Joint Infections in Children. J Am Acad Orthop Surg.

[10] Kasper DC, Altiok I, Mechtler TP, Böhm J, Straub J, Langgartner M (2013). Molecular detection of late-onset neonatal sepsis in premature infants using small blood volumes: proof-of-concept. Neonatology.

[11] Van Hal SJ, Lodise TP, Paterson DL (2012). The clinical significance of vancomycin minimum inhibitory concentration in Staphylococcus aureus infections: a systematic review and meta-analysis. Clin Infect Dis.

[12] Kullar R, Davis SL, Levine DP, Rybak MJ (2011). Impact of vancomycin exposure on outcomes in patients with methicillin-resistant Staphylococcus aureus bacteremia: support for consensus guidelines suggested targets. Clin Infect Dis.

